# The role of multimodality imaging in selection, response assessment, and follow-up of patients receiving ^177^Lutetium-PSMA-therapy

**DOI:** 10.1186/s13244-025-02151-x

**Published:** 2026-01-16

**Authors:** Aditi Ranjan, Minal Padden-Modi, Hoda Abdel-Aty, Joao Galante, Simon Wan, Azzra Maricar, Adetokunbo Adesina, Brent Drake, Siraj Yusuf, Gary Cook, Nicholas James, Sola Adeleke

**Affiliations:** 1 Oxford University Hospitals NHS Foundation Trust, Oxford, UK; 2 Institute of Cancer Research and Royal Marsden Hospital NHS Foundation Trust, London, UK; 3 Oncology, Guy’s and St Thomas’ NHS Foundation Trust, London, UK; 4 Institute of Nuclear Medicine, University College London Hospitals NHS Foundation Trust, London, UK; 5 King’s College London, London, UK; 6 Buckinghamshire Hospitals NHS Trust, Amersham, UK; 7 Radiology and Nuclear Medicine Department, Royal Marsden NHS Foundation Trust, London, UK; 8 King’s College London, Cancer Imaging PET Centre, St Thomas’ Hospital, London, UK; 9 KCL, School of Biomedical Engineering and Imaging Sciences, Becket House, London, UK

**Keywords:** MRI-diffusion-weighted imaging, PSMA-PET/CT, Molecular imaging–cancer, Prostate

## Abstract

**Abstract:**

Prostate cancer is the most commonly diagnosed cancer among men in 112 countries, accounting for approximately 15% of all cancer cases. Whilst the 5-year survival rate for localised disease exceeds 90%, there is a significant drop to 50% if metastases are present. Following the VISION and TheraP trials, ^177^Lu-PSMA-therapy was approved for treatment of metastatic castrate resistant prostate cancer by the FDA and EMA 2022. Patient selection for ^177^Lu-PSMA-therapy is now relatively well defined, guided by PSMA-PET/CT criteria established in pivotal trials. Nevertheless, clinical consensus on appropriate criteria is still evolving, and additional imaging modalities such as ^18^F-FDG PET, post-therapy SPECT/CT, or emerging techniques such as whole-body diffusion-weighted MRI may serve as valuable adjuncts to identify PSMA-negative or treatment-resistant disease that may not be apparent on PSMA-PET/CT alone. This review examines the current evidence on imaging biomarkers and complementary diagnostic techniques used for patient selection, treatment monitoring, and response assessment in [¹⁷⁷Lu]Lu-PSMA-617 therapy for metastatic castrate resistant prostate cancer. Baseline imaging biomarkers on PSMA-PET/CT, such as mean standardised uptake value (SUV_mean_), PSMA-avid total tumour volume, and inter-lesional PSMA heterogeneity, have shown promise in predicting treatment response and assessing outcomes. Additionally, statistical prognostic models have been developed to predict treatment efficacy, though further validation is required. Imaging plays a crucial role and should be considered alongside blood biomarkers, clinic-demographic history, and circulating tumour markers to improve patient selection for ^177^Lu-PSMA-therapy.

**Critical relevance statement:**

PSMA-PET/CT is the established imaging modality for patient selection for ¹⁷⁷Lu-PSMA-therapy, while ¹⁸F-FDG PET, post-therapy SPECT/CT, and emerging techniques such as whole-body diffusion-weighted MRI can be adjunctive for patient selection, response assessment and long-term monitoring.

**Key Points:**

- PSMA-PET/CT is the mainstay for patient selection for ¹⁷⁷Lu-PSMA-therapy. ^18^F-FDG PET, SPECT/CT or whole-body diffusion-weighted MRI could be used as adjuncts.
- Interim and longitudinal PSMA-PET/CT offer sensitive detection of progression, quantitative biomarkers for response assessment, and standardised frameworks.
- Advances in AI, radiomics, and standardisation frameworks may refine prognostication, enable personalised dosimetry, and integrate imaging biomarkers into clinical practice, though further validation is required.

**Graphical Abstract:**

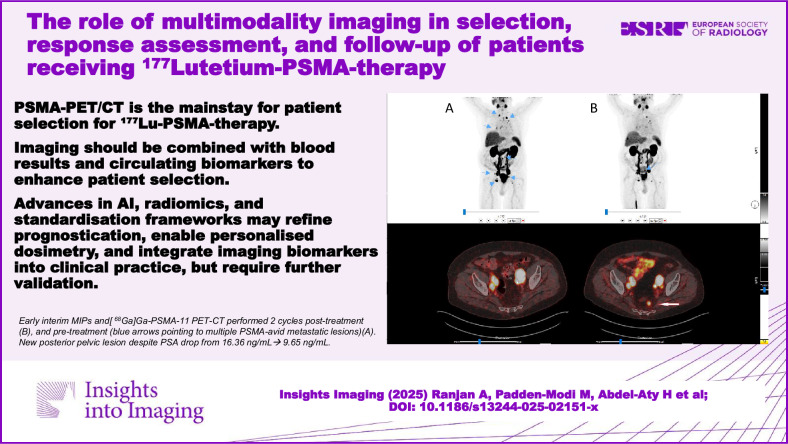

## Background

Prostate cancer (PCa) is the most frequently diagnosed cancer among men in 112 countries, accounting for 15% of all cancer cases. Globally, there are approximately 1.4 million new cases and 375,000 deaths each year, making it the sixth leading cause of cancer death in men [1]. The 5-year survival exceeds 90% for localised disease, dropping to 50% if metastases are present, highlighting the need for improved treatments for advanced PCa [2].

**Fig. 1:**
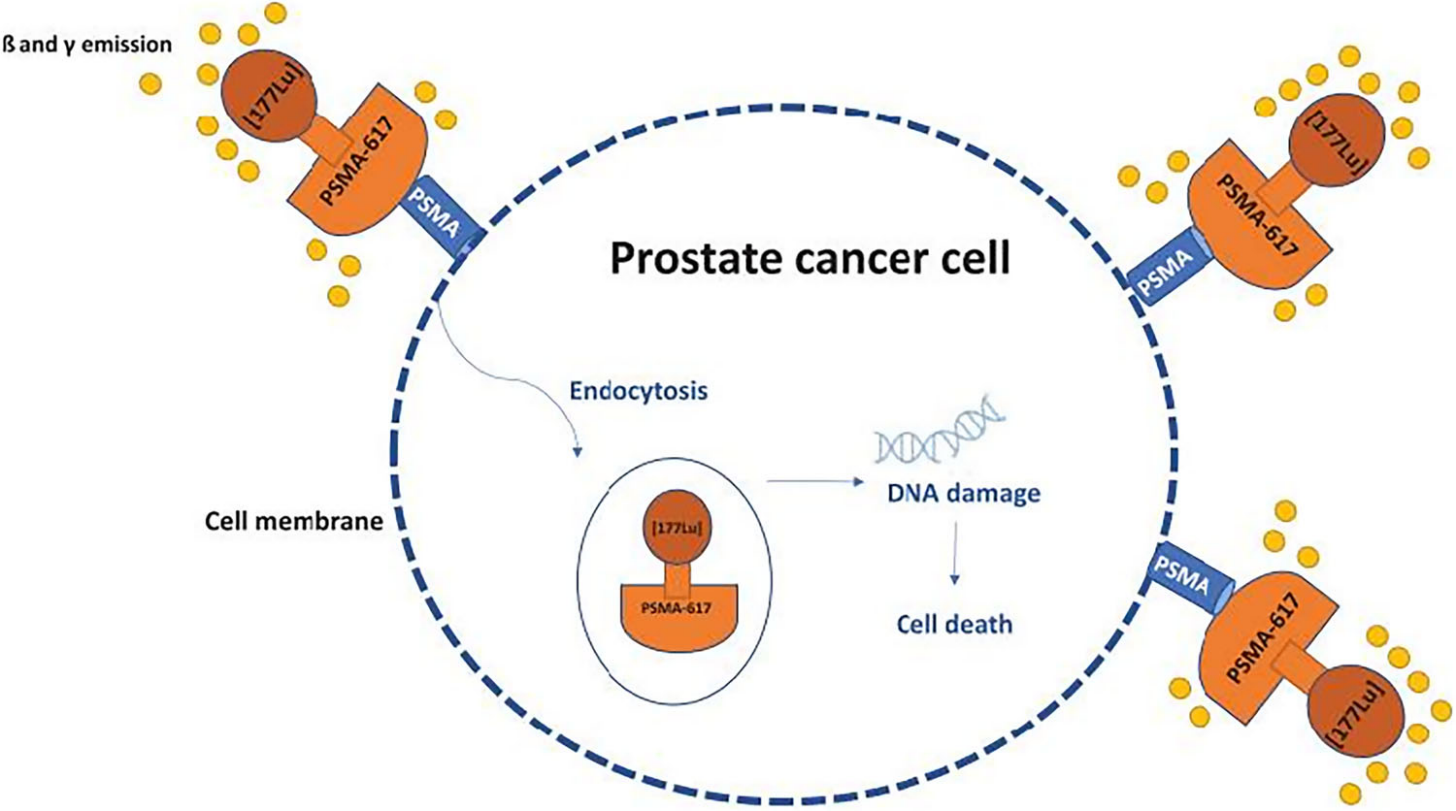
Mechanism of action of ^177^Lu-RLT. Courtesy of Heidegger I [55]

Treatment options for metastatic castration-resistant prostate cancer (mCRPC) include chemotherapy (e.g., docetaxel, cabazitaxel), androgen receptor pathway inhibitors (ARPIs, e.g., enzalutamide), poly(adenosine diphosphate-ribose) polymerase inhibitors for those with DNA deficiency repair mutations (e.g., olaparib) and radioligand therapies (RLT) such as Radium-223 and ^177^Lu-PSMA-Therapy [1, 3]. The latter targets prostate-specific membrane antigen (PSMA), a molecule overexpressed in PCa cells, enabling its use in both imaging and treatment [4]. The mechanism of action of ^177^Lu-PSMA-therapy is illustrated in Fig. 1.

### Evidence for ^177^Lu-PSMA-therapy

The VISION phase III trial randomised participants 2:1 to receive either six cycles of ^177^Lu-PSMA-therapy plus standard of care (SOC), versus SOC alone. ^177^Lu-PSMA-therapy improved overall survival (OS) (15.3 months vs. 11.3 months), and delayed radiographic progression-free survival (rPFS) when added to SOC (median rPFS for patients treated with ^177^Lu-PSMA was 8.7 vs. 3.4 months) [5]. The TheraP phase II trial compared [^177^Lu]Lu-PSMA-617 to cabazitaxel, and found superior PSA50 responses with [^177^Lu]Lu-PSMA-617 (66% vs. 37% achieving ≥ 50%PSA decline) [6]. Furthermore, patients with higher average maximum standardised uptake value (SUV_max_) on PSMA-PET/CT had higher PSA response rates to [^177^Lu]Lu-PSMA-617. The PSMAfore trial evaluated patients with PSMA-positive mCRPC progressing on at least ARPI, and demonstrated a 59% reduction in the risk of disease progression compared to an alternative ARPI and more than doubled median rPFS compared to a switch in ARPI (11.6 vs. 5.6 months) [7]. The EnzaP trial is a Phase II study investigating the combination of [177Lu]Lu-PSMA-617 with enzalutamide vs. enzalutamide alone in mCRPC patients post-docetaxel. The combination arm showed superior PSA50 response rates and rPFS, supporting the potential of synergistic combinations involving RLT and androgen receptor targeted therapies [8]. Collectively, these trials highlight the role of PSMA-targeted RLT in mCRPC and its emerging potential in earlier metastatic castrate-sensitive disease.

### Aims

[^177^Lu]Lu-PSMA-617 was approved by the U.S. Food and Drug Administration and European Medicines Agency in 2022 for post-taxane mCRPC, and in 2025 for pre-chemotherapy PSMA-positive mCRPC progressing following treatment with a single ARPI, based on the Phase III PSMAfore trial. The eligible patient population is estimated to triple as a result [9]. Currently, evidence for optimal patient selection for ^177^Lu-PSMA-Therapy for mCRPC is limited, with no standardised criteria. The substantial costs of ^177^Lu-PSMA-Therapy and limited radiotracer availability highlight the need to optimise patient selection and response assessment tools to minimise financial harm, for equity of access, and for sustainable adoption of RLT.

This review examines the role of imaging and circulating biomarkers in guiding patient selection, response evaluation, and long-term monitoring for ^177^Lu-PSMA-Therapy for mCRPC. It highlights the established PSMA-PET/CT, emerging modalities such as whole-body MRI, somatostatin receptor PET/CT, and quantitative dosimetry, alongside integration of imaging biomarkers, radiomics, AI, and composite models to inform personalised treatment strategies.

## Baseline patient selection

### Contrast-enhanced CT and bone scintigraphy (BS)

Imaging with CT and BS continues to play a role in clinical trials; for instance, all patients in the VISION study were required to have mCRPC confirmed with CT and BS. However, there are diagnostic limitations: CT detects non-localised disease with only 40% sensitivity, while BS has a cumulative sensitivity of ~80% [10, 11]. CT performs particularly poorly in detecting micrometastatic nodal involvement, and BS is prone to specificity issues, as it frequently detects benign degenerative changes alongside malignant lesions [12]. Its sensitivity is also highly dependent on prostate-specific antigen (PSA) level: for PSA < 20 ng/mL, the sensitivity for skeletal metastases compared with PSMA-PET/CT is only 46%, increasing to 89% for PSA > 20 ng/mL. However, BS cannot identify soft-tissue disease such as nodal or visceral metastases, reducing sensitivity in high-risk patients (PSA > 20 ng/mL) to only ~50% [13]. Data from the proPSMA trial further shows CT and BS to have a sensitivity of less than 50% when compared directly to PSMA-PET/CT [14]. Conventional imaging remains a cost-effective and accessible alternative in settings where PSMA-PET tracers are not available. In resource-rich settings, conventional imaging can play a complementary role alongside PSMA-PET/CT, for example, in identifying PSMA-negative disease or complications not well visualised on PET.

### PSMA-PET/CT

PSMA-PET/CT imaging has high sensitivity and specificity for bone, nodal, and soft-tissue metastases compared to CT and BS [15, 16]. PSMA-PET identifies more sites of metastatic disease, often reclassifying patients from low to high-volume disease; therefore, stratification based on conventional imaging may misrepresent true disease burden [17]. Accurate detection of PSMA-avid disease is essential for determining eligibility for ¹⁷⁷Lu-PSMA-therapy.

PSMA-PET/CT provides quantitative biomarkers through SUV such as SUV_max_, SUV_peak_, PSMA-avid tumour volume (PSMA-TV) and total lesion PSMA expression (TL-PSMA). Higher baseline SUV_mean_ has been associated with better outcomes. In TheraP, patients with an SUV_mean_ ≥ 10 had a significantly higher PSA response rate to [^177^Lu]Lu-PSMA-617 than to cabazitaxel (91% vs. 47%) [6]. VISION also found that each unit increase in total tumour SUV_mean_ reduced rPFS risk by 12% and mortality by 10% [5]. PSMA-TV has similarly shown strong predictive and prognostic value [18]. In LuPIN, while a higher SUV_mean_ was linked to treatment response, lower PSMA-TV and longer ARPI exposure were associated with worse OS [19].

Despite its advantages, the clinical application of SUV_mean_ is limited by resource-intensive total-body image quantification. Recent work has identified the heterogeneity and intensity tumour (HIT) score as a promising alternative, through visual assessment of tumour heterogeneity on standard clinical PET workstations. The HIT score showed predictive value for PSA50 response, PSA-PFS, and OS, comparable to SUV_mean_ quartiles, with patients with the highest HIT scores having better outcomes compared to those with the lowest scores (76% vs. 0% PSA50, 8.5 vs. 1.0 months PSA-PFS, and 16.9 vs. 7.6 months OS) [20].

Parotid PSMA uptake is generally higher than the liver, and can be used as a visual reference organ. The whole-body tumour-to-parotid SUV_mean_ ratio can be utilised as a biomarker, with higher ratios correlating with greater PSA50 response rates (63% vs. 17%) and longer PSA-PFS and OS (6.7 vs. 1.9 months and 14.3 vs. 12.9 months, respectively). This metric is reproducible, can be visually estimated from maximum intensity projections, and indirectly reflects tumour heterogeneity, highlighting its potential utility as a stratification tool [21].

PSA change remains the key predictor of OS. Iravani et al showed that pretreatment SUV_mean_ correlated with tumour-absorbed radiation dose, and was prognostic for OS, with lower uptake (< 10 Gy) linked to weaker PSA responses [22]. Conversely, Ferdinandus et al suggest PSA response does not always align with PSMA uptake, highlighting the complexity of treatment response relating to potential biological factors [23]. Emerging evidence supports early changes in PSMA-TL as a superior survival marker compared to PSA [24]. Still, VISION data confirmed that PSA decline remains clinically meaningful, correlating with rPFS, OS, and improved QOL [25]. Additional predictive factors include: maximal PSMA intensity (tumour-to-background uptake in the highest SUV_mean_ lesion), and PSMA tumour heterogeneity on baseline ^68^Ga-PSMA-PET/CT. Both correlated with improved therapeutic outcomes and could optimise patient selection [24].

[^177^Lu]Lu-PSMA-PET/CT enables standardised stratification through the Prostate Cancer Molecular Imaging Standardised Evaluation Framework Including Response Evaluation for Clinical Trials (PROMISEV2) criteria by assessing PSMA expression intensity and lesion distribution: identifying miPSMA-positive lesions ensures only patients with sufficient expression are selected for RLT, while metrics such as SUV_mean_, SUV_max_ and HIT score correlate with treatment response and survival [26].

Most trials rely only on [^68^Ga]Ga-PSMA-11 PET/CT to identify suitable participants, such as the VISION trial, excluding 13% with PSMA-negative lesions [5]. Yet, despite imaging-based selection, there was a 51% response rate with approximately one-third of men in VISION not achieving PSA50, highlighting the need for refined patient selection [5, 27]. VISION data showed 86.6% of patients would have been eligible for ^177^Lu-PSMA-therapy without pre-screening PSMA-PET/CT, highlighting the limitations of PET/CT as a sole predictive tool [17]. Patients with low PSMA expression, particularly those with PSMA-negative but FDG-avid disease, may derive greater benefit from chemotherapy: trials combining [^177^Lu]Lu-PSMA-617 followed by pembrolizumab, using ^68^Ga-PET/CT and/or [^18^F]FDG PET, have demonstrated anti-tumour activity even in low or PSMA-negative disease [28, 29].

Debate continues regarding whether mandatory screening with PSMA-PET/CT and contrast-enhanced CT is necessary, especially since the VISION trial would have possibly yielded positive results even without excluding any patients. Conversely, proponents of multimodal imaging, including dual-tracer PET (PSMA and FDG) or even triple-tracer approaches (PSMA, FDG, and somatostatin receptor (SSTR) imaging), argue that multimodal screening helps to refine patient selection to those who will benefit most in cases of ^177^Lu-PSMA. Treatment is associated with side effects such as renal toxicity and xerostomia. This approach may be valuable in settings with limited ¹⁷⁷Lu-PSMA-supply, particularly in low- and middle-income countries. Nevertheless, expanded imaging burden results in financial toxicity (with treatment costs reaching $300,000 for a six-cycle course) and logistical challenges.

### FDG PET/CT

Imaging with ¹⁸F-FDG PET/CT is prognostically useful, with intraprostatic uptake linked to aggressive disease due to elevated glucose metabolism [30]. Whilst not widely used in earlier disease due to PSMA-PET/CT (given superior sensitivity and specificity), ¹⁸F-FDG PET/CT is a valuable adjunct for identifying PSMA-low disease or PSMA-negative disease. In the UpFrontPSMA trial, 21% of patients were not eligible due to discordant disease (FDG-positive/PSMA-negative lesions) [31]. One-third of screened patients were excluded from TheraP for this reason. While significantly higher PSA response rates were observed with [^177^Lu]Lu-PSMA-617 versus cabazitaxel (66% vs. 37%, *p* < 0.0001), high FDG metabolic tumour volume (MTV ≥ 200 mL) was associated with poorer outcomes across arms on post-hoc analysis, suggesting the FDG-high cohort may require more intensive treatment strategies [6, 32].

Conversely, VISION employed broader eligibility criteria with lower PSMA-uptake thresholds and omitted FDG PET. A retrospective comparison of TheraP and VISION showed patients meeting the stricter TheraP standards had higher PSA response rates and improved PFS, and patients with discordant FDG-positive/PSMA-negative disease had poorer outcomes [33]. Conversion from PSMA-positive to PSMA-negative phenotype has also been reported as a resistance mechanism [34]. These findings support incorporating FDG PET to refine patient selection. Whilst being complementary to PSMA-PET/CT, its use comes with added cost, radiation exposure, and logistical challenges. Currently, there is no consensus on utilising FDG PET for patient selection, with trials like VISION omitting it entirely. Its use is currently centre-specific, and further validation is needed to establish standardised protocols.

### ^18^F-PSMA-PET/CT

Many ^18^F-labelled radiotracers (such as [^18^F]DCFPyL) have a comparable biodistribution to ^68^Ga, and can be an alternative. ^18^F-labelled radiotracers offer practical advantages, including a longer half-life (enabling broader distribution to PSMA-imaging centres) and cyclotron-based production. Unlike most ^18^F-labelled radiotracers, [^18^F]F-PSMA-1007 undergoes hepatobiliary clearance rather than renal clearance, improving lesion detection near the bladder and pelvic sidewalls [35, 36].

Comparative studies have found higher SUV_max_ values and improved tumour-to-background ratios with [^18^F]DCFPyL compared to [^68^Ga]Ga-PSMA-11, detecting additional sites of disease and highlighting its potential for increased sensitivity in detecting small-volume disease [37]. Overall, [^18^F]DCFPyL is deemed to be a suitable alternative to [^68^Ga]Ga-PSMA-11 in diagnosis and staging [36].

However, non-specific bone uptake and benign PSMA-avid findings, particularly in ribs with [^18^F]F-PSMA-1007, can complicate interpretation [35, 36]. A 2024 meta-analysis found [^18^F]F-PSMA-1007 to be associated with a higher prevalence of equivocal bone lesions compared with [^68^Ga]Ga-PSMA-11 and [^18^F]DCFPyL (36% vs. 8%), with a lower malignancy rate (8% vs. 29%) [38]. A systematic review also found unspecific bone uptakes (UBUs) were most frequent with [^18^F]F-PSMA-1007, and their topographical distribution (rib uptake being rarely metastatic) can help guide interpretation [39].

Whilst ^18^F-labelled radiotracers offer logistical and potential imaging advantages, most guidelines recommend using the same radiotracer consistently across a patient’s disease course, and caution when interpreting bone findings with [^18^F]F-PSMA-1007, as there is a potential for false positives and over-staging.

### ^99m^Tc-PSMA-SPECT/CT

^99m^Tc-labelled SPECT/CT is a more accessible and cost-effective alternative to [^68^Ga]Ga-PSMA-PET/CT, with comparable diagnostic performance [40]. ^99m^Tc-HYNIC-PSMA-SPECT has shown superior detection of osseous lesions over PSMA-PET/CT, identifying 34.5% of bone lesions missed by other modalities [41]. Its long half-life, PSMA-avidity, and improved reconstruction techniques contribute to its utility, particularly in bone metastases [42]. However, one study noted reduced lesion detection with ^99m^Tc-PSMA-SPECT/CT in the prostate bed in a comparative analysis [43].

Although PSMA-PET/CT offers superior spatial resolution and sensitivity, its widespread use is limited by costs, radiotracer availability, and infrastructure requirements. In contrast, ^99m^Tc-PSMA-SPECT/CT is more accessible globally, due to lower operational costs and broader availability of SPECT scanners. In resource-limited settings where PET/CT is not readily available, ^99m^Tc-PSMA-SPECT/CT can be an effective alternative for disease staging and treatment planning, particularly in patients with a high burden of osseous metastases.

Beyond diagnostics, SPECT/CT plays a key role in the post-therapeutic setting, with post-treatment dosimetry, therapy verification, and early assessment of treatment response. These highlight the role of SPECT/CT in the evaluation of the efficacy of treatment.

### Liquid biopsies

Circulating tumour DNA (ctDNA) analysis offers additive prognostic insight beyond PSA and imaging: in a post-hoc analysis of the TheraP trial, patients with low ctDNA% had a 100% biochemical response rate and a markedly longer median PFS (14.7 vs. 6.0 months) with [¹⁷⁷Lu]Lu-PSMA-617, independent of PSMA-PET imaging parameters. This underscores ctDNA% as an imaging-independent biomarker for identifying patients most likely to benefit from PSMA-RLT. Moreover, in patients with higher ctDNA% ≥ 2%, the presence of PTEN alterations was associated with a significantly improved PSA50 response and PFS, and deleterious alterations in DNA damage repair genes (such as BRCA2 and ATM) were associated with longer responses. This highlights the potential role of ctDNA in identifying molecular subgroups that would gain the greatest benefit from PSMA-RLT [44].

Reductions in tumour volume and ctDNA at 12 weeks correlated with better response [27]. The PSA flare phenomenon (characterised by a transient PSA rise before response), can occur up to 12 weeks after therapy initiation–a post-hoc analysis of the VISION trial found some patients with early PSA increases eventually show partial or complete response, indicating that a flare does not preclude good patient outcomes [25]. Emerging biomarkers include cell-free (cf) DNA epigenomic signals at the FOLH1 (PSMA) locus: acting as a surrogate for tumour PSMA expression, correlated highly with total-tumour PSMA-PET/CT SUV_mean_ (*p* < 0.05), and was potentially a predictive biomarker of therapeutic response [45].

## Interim and post-treatment response assessment

### Interim PSMA-PET/CT

**Fig. 2:**
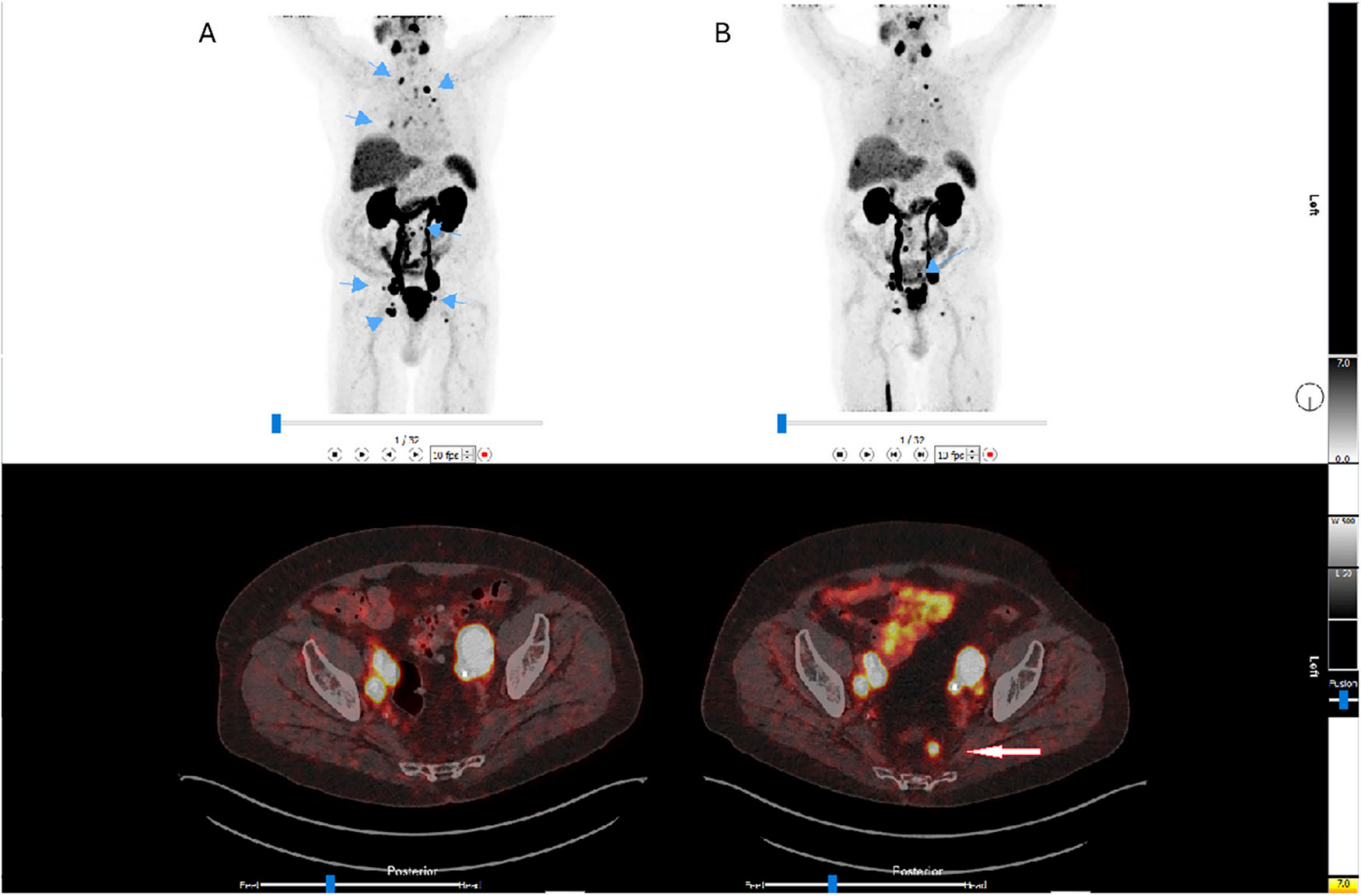
Early interim MIPs and [^68^Ga]Ga-PSMA-11 PET-CT performed 2 cycles post-treatment (blue arrow points to a reduced area of radiotracer uptake, suggesting partial metabolic response or residual disease at that site. Most previously PSMA-avid lesions have resolved or show significantly lower uptake (**B**), and pretreatment (blue arrows pointing to multiple PSMA-avid metastatic lesions) (**A**). New posterior pelvic lesion despite PSA drop from 16.36 ng/mL to 9.65 ng/mL. Courtesy of Drake B

Response to ^177^Lu-PSMA-Therapy is typically monitored using PSA levels, CT, and bone scintigraphy, according to Response Evaluation Criteria in Solid Tumours (RECIST1.1) and Prostate Cancer Working Group (PCWG) 3. However, seroconversion to non-PSA-secreting disease and osteoblastic changes can complicate interpretations under PCWG3 criteria. The PCWG3 criteria standardise definitions for biochemical and radiographic progression in PCa using PSA kinetics, soft tissue changes on CT, and BS, whereas RECIST1.1 relies on anatomical size changes on CT or MRI to classify treatment response in measurable soft-tissue disease. Kleiburg et al evaluated PSMA-PET/CT response in patients following ARPI or chemotherapy, finding that PSMA-PET/CT outperformed PSA as an independent predictor of OS, discordance between PSA and PSMA-PET/CT was demonstrated in 47% of patients, and 31% of patients achieving PSA50 showed disease progression on PET/CT, highlighting the limitations of PSA alone for assessing treatment benefit [46]. PSMA-PET/CT can detect sub-centimetre lesions with higher sensitivity and a favourable tumour-to-background ratio, and is therefore increasingly used to assess treatment response (Fig. 2) [47]. Consequently, several criteria have been proposed for detecting biochemical recurrence, such as the PSMA PET progression (PPP) criteria (appearance of new PSMA-avid lesions), the Response Evaluation Criteria in PSMA-PET/CT (RECIP) 1.0 (PSMA-PET/CT specific framework evaluating percentage change in total PSMA-positive TTV and appearance of new lesions to classify patients as responders, stable, or progressing), and PROMISEV2 (integration of molecular imaging TNM (miTNM) staging, PSMA-expression score, and anatomical reporting of lesion distribution) [26, 48, 49].

PSMA-PET/CT has emerged as a valuable tool for interim response assessment, compared to PSA or size-based CT criteria. One consideration is tumour burden at baseline: in patients with high-volume metastatic disease, small new lesions on post-treatment PSMA-PET/CT may be difficult to interpret. European Association of Urology (EAU) and the European Association of Nuclear Medicine (EANM) define non-response on post-treatment PET/CT differently depending on baseline disease burden: an increase in total tumour volume (TTV) > 30% is required to classify polymetastatic disease as ‘non-responding’, whereas appearance of > 2 new lesions indicates non-response in low-burden cases [50, 51].

The PET Response Criteria in Solid Tumours (PERCIST1.0) assesses metabolic response using SUV changes in the most metabolically active lesion (‘hottest lesion’) to evaluate early molecular response before anatomical changes [52]. Unlike RECIST 1.1, PCWG3, PET Response Criteria in Solid Tumours (PERCIST), or PPP criteria, RECIP integrates both the occurrence of new lesions and changes in PSMA-avid TTV in defining progression. The RECIP classification relies on quantitative whole-body segmentation software to measure TTV, which is not yet widely available for clinical practice. Recent studies have shown qualitative visual RECIP reads to have good concordance with quantitative RECIP (κ = 0.89) and strong inter-reader agreement in distinguishing progressive disease from non-progressive disease, identifying patients unlikely to benefit from treatment. Visual RECIP classifications are also associated with OS [49]. Automated algorithms have been developed to assign RECIP classifications to post-therapy PSMA-PET/CTs, and can match manual RECIP classifications in 97% of cases. This algorithm can also track individual lesions from baseline imaging and detect new sites of disease [53]. Quantitative RECIP has superior reproducibility and more consistent inter-reader agreement than visual reads, and will likely become standard once segmentation tools are validated. Quantitative PSMA-PET biomarkers such as PSMA-VOL and TL-PSMA have demonstrated strong predictive value for OS, outperforming PSA for response assessment [26, 54]. Interim scans after two treatment cycles offer prognostic insight: persistent PSMA-avid disease or limited reduction in PSMA-TV is associated with poorer outcomes, with end-of-treatment PSMA-PET after the last therapy cycle being associated with OS [49, 55–58]. Rosar et al found that TL-PSMA, assessed after two cycles of ^177^Lu-PSMA therapy using PERCIST 1.0 criteria, was an independent predictor of OS, outperforming PSA-based response evaluation [54]. PSMA-PET/CT volumetric measures not only reflect biochemical response, but also provide a more sensitive metric for early treatment response, particularly in heterogeneous metastatic disease where morphological changes may lag behind functional activity.

Post-radiotherapy PSMA-PET detects residual intraprostatic disease in up to 39% of patients, and new pelvic or extra-pelvic lesions in approximately 40%, even at low PSA levels [59]. Post-radiotherapy PSMA-PET signal is influenced by both lesion site and timing of imaging. A retrospective study of 217 irradiated lesions in 89 patients showed PSMA-lesion uptake to decline over time, with the lowest SUV_max_ at 9–12 months post-radiotherapy, with residual uptake more common in the prostate/prostate bed and in lesions with higher baseline SUV_max_. These findings highlight that early PSMA-PET imaging may overestimate residual disease, and the importance of timing for assessing treatment response to avoid misinterpretation of early uptake as persistent disease [60]. Higher PSA values (> 10 ng/mL) are associated with a greater risk of distant metastases. However, PSMA-PET interpretation in the early post-radiation period (< 1 year) remains challenging due to the risk of false positives from inflammatory changes or indolent residual disease [59]. Despite this, PSMA-PET remains valuable as an early interim marker of benefit, guiding salvage interventions, particularly metastasis-directed therapy or targeted re-irradiation when early limited recurrence is identified [50]. Quantitative PET parameters such as SUV_max_, SUV_mean_, and SUV_peak_ are key when it comes to response evaluation. However, their reliability depends on standardisation of radiotracer injection, scanner type, and reconstruction algorithms, to ensure reproducibility.

PSMA expression increases following ARPI, especially in mCRPC. This has been used to leverage trials such as ENZA-P, where ^177^Lu-PSMA-Therapy and enzalutamide are given synergistically [8]. PSMA-PET/CT may identify suitable patients for ^177^Lu-PSMA-Therapy who have PSMA-flare, as patients demonstrating an increase in SUV on PSMA-PET/CT 15 days after commencing ARPI had the largest depth and duration of PSA response in ENZA-P translational work [61].

### Post-therapy ^177^Lu-SPECT/CT

While not used for initial patient selection, post-therapy SPECT/CT is often performed 24–72 h post-administration. Although PET/CT has higher spatial resolution, allowing evaluation of sub-centimetre lesions, SPECT/CT can assess tumour burden without additional radiation as it utilises the therapeutic isotope, and SPECT/CT-capable gamma-cameras are more widely available (Table 1) [62].

**Table 1:** Comparison of PSMA-PET/CT and SPECT/CT for interim response assessment

Imaging modality	PSMA-PET/CT	SPECT/CT
Timing	• Typically after 2 cycles of ¹⁷⁷Lu-PSMA therapy • Can be done at end-of-treatment	• 24–72 h post-therapy (can be as early as 1–5 h) • Repeated after cycles 1–2.
Radiotracer	Requires diagnostic PSMA ligand (e.g., [⁶⁸Ga]Ga-PSMA-11, [¹⁸F]F-DCFPyL).	Utilises therapeutic radionuclide (¹⁷⁷Lu), no additional tracer needed.
Spatial resolution/sensitivity	High spatial resolution, detects sub-centimetre lesions	Lower spatial resolution, cannot reliably detect small lesions.
Quantitative biomarkers	• Volumetric parameters: PSMA-VOL, TL-PSMA (associated with PFS and OS) • SUVmax, SUVmean, SUVpeak • PET/CT derived TTV changes	• SPECT-derived TTV changes (associated with PSA-PFS and OS) • New lesion appearance
Interpretation frameworks	• RECIP 1.0 (integrates new lesions + TTV changes) • PROMISE V2 • PPP	• No universally accepted response criteria, prognostic thresholds vary across studies.
Practical considerations	• Requires radiotracer synthesis • Requires PET scanner access • More costly, less widely available • Standardised acquisition and reconstruction	• Widely available γ-cameras, useful in resource-limited settings • Cost-effective • No additional radiation
Limitations	• Interpretation challenging in polymetastatic disease • Risk of false positives post-radiotherapy	• Lower resolution may underestimate small-volume disease • Lack of standardisation across centres
Clinical utility	• Early detection of treatment failure • Can guide adaptive treatment strategies • Can be used for clinical trial stratification	• Real-time information on tracer delivery and dosimetry • Early marker of non-response – can guide management changes

Early changes in SPECT-derived TTV are strongly prognostic: reductions in TTV between first and second cycles predicted longer PSA-PFS, while a > 30% decline after cycle 2/3 was associated with a survival benefit (median OS not reached vs. 6 months, PSA-PFS 6 vs. 1 month) [20, 63, 64]. Conversely, TTV increased by week 6 correlated with significantly shorter PSA-PFS (3.7 vs. 6.7 months). Notably, the combination of early PSA rise and TTV progression identified a subgroup with particularly poor outcomes (median PSA-PFS 2.8 vs. 9.0 months) [65]. Moreover, the appearance of new lesions on serial SPECT/CT was an independent adverse prognosticator, with HRs for death of 5.8 at cycle 2 and 4.9 at cycle 3, independent of PSA changes [66]. Demirci et al found that new lesions or increased tumour volume detected on SPECT/CT 24 h post-therapy were independently linked to higher mortality and led to management changes in 49% of cases in a study by Yadav et al [66, 67]. Similar results have been observed in studies using [^177^Lu]Lu-PSMA-I&T, and in the LuPIN trial combining [^177^Lu]Lu-PSMA-617 with NOX66 [68, 69]. Additional evidence supports these findings, with John et al reporting that SPECT-derived TTV after 2 cycles predicted PFS but not OS, while Song et al found ultra-early (1–5 h) post-therapy SPECT to predict OS [63, 69]. Neubauer et al found that changes in SPECT-derived TTV after 2 cycles were independently associated with OS, strengthening the use of SPECT/CT as a prognostic biomarker across different cohorts [70]. SPECT-derived TTV and new lesion detection are early, non-invasive markers of treatment response and survival, allowing for early treatment modification for non-responders, avoiding unnecessary treatment toxicity. Beyond prognostication, post-therapy SPECT/CT can also be used to personalise treatment: serial SPECT-derived metrics can identify patients who will not benefit from ^177^Lu-PSMA-Therapy as early as after the second cycle, allowing timely therapy modification. Furthermore, SPECT/CT-guided assessment of TTV and lesion progression has been shown to alter management in up to 49% of patients, highlighting its role as an imaging-based biomarker of response [62].

EANM guidelines recommend scintigraphy or SPECT/CT 1–2 days post-administration to assess RLT delivery and organ dosing [34]. Whilst it can allow early detection of non-responding or non-avid lesions and assist with dosimetry, ¹⁷⁷Lu-SPECT/CT is not standardised for interim response assessment across centres. It could play a complementary role in confirming radiotracer targeting and supporting treatment planning in resource-conscious settings.

### Contrast-enhanced CT/bone scintigraphy

CT and BS remain central to assessing disease progression and treatment response under PCWG3 criteria, including the definition of rPFS, which correlates strongly with OS in men with mCRPC [71]. CT is supported by standardised acquisition protocols and, combined with RECIST1.1, provides a reproducible and comparable tool for both clinical trials and practice [72].

**Fig. 3:**
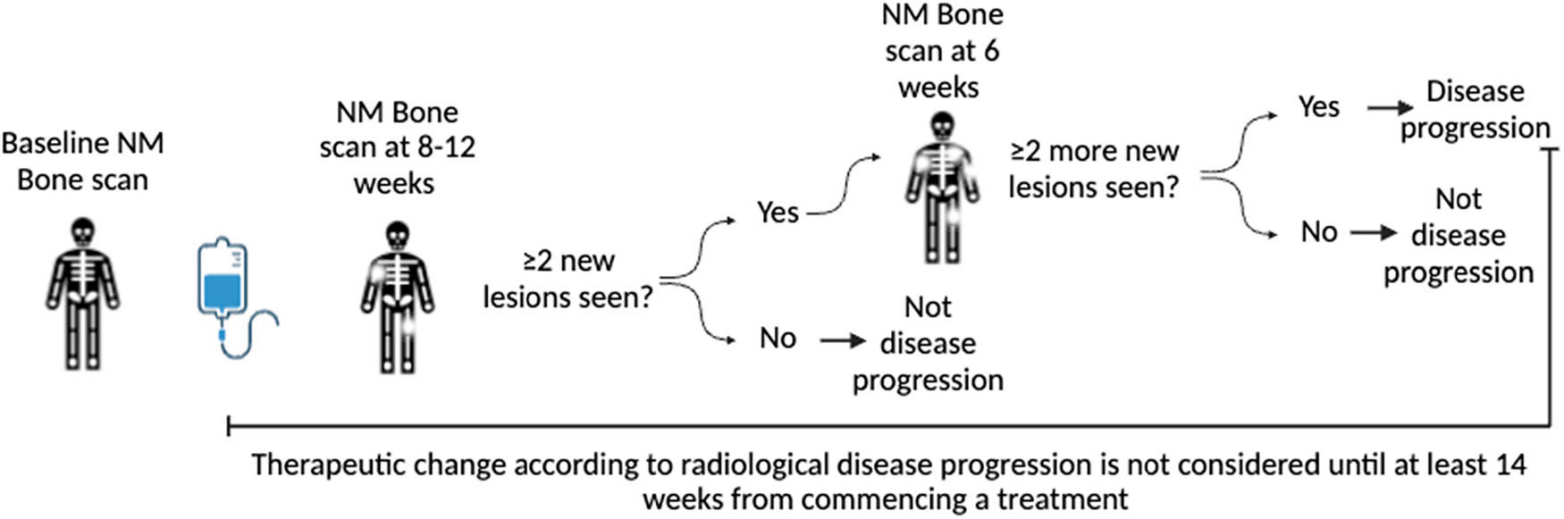
PCWG3 criteria flowchart (courtesy of Padden-Modi M)

Most trials utilise PCWG3, assessing soft tissue disease via RECIST1.1, and bone lesions using a 2 + 2 rule to account for flare at 8 weeks on bone scintigraphy (Fig. 3) [72]. The 2 + 2 rule requires the appearance of at least two new lesions on BS to be confirmed by two additional new lesions on a subsequent scan before concluding progression, reducing the risk of misclassifying healing responses as disease progression. However, PCWG3 criteria require a 16-week interval before changing ineffective therapies, and cannot identify lytic bone metastases. Furthermore, he 2 + 2 rule may misclassify progression as stable disease if only one new lesion develops post-flare [72]. The RECIST1.1 criteria also have limitations: lymph nodes < 1.0 cm in short axis are not measurable on CT, potentially missing smaller metastases visible on PSMA-PET/CT. Although RECIST1.1 is strongly correlated with OS, its applicability in PCa is limited due to predominantly sclerotic bone lesions. PCWG3 emphasises the need for more sensitive imaging techniques to assess treatment response and progression. The challenge is compounded by bone scan flare, which can cause difficulty distinguishing healing from true progression.

Trials such as STAMPEDE2 evaluated soft-tissue disease using RECIST 1.1 and bone metastases via the 2 + 2 rule to account for the bone scan flare [73]. However, data using PSMA-PET as the reference standard have shown BS to have low positive predictive value (PPV 0.43) and frequently overestimate metastatic burden at initial staging, with over half of patients misclassified as having osseous disease [74]. These findings suggest that disease-volume thresholds from trials using conventional imaging may not translate to PSMA-PET staged cohorts, where lesions are detected with higher sensitivity and inter-reader agreement (κ = 0.80 vs. 0.51 for BS). While PCWG3 and the 2 + 2 rule remain important for continuity with historical trial endpoints, PSMA PET/CT more accurately measures metastatic burden.

Nikitas et al found that BS and CT and PSMA-PET/CT detected similar numbers of bone lesions in the majority of patients, though PET/CT identified additional lesions in 27% of cases, highlighting its higher sensitivity. However, evidence increasingly favours PSMA-targeted imaging: Holzgreve et al found metastatic disease in 46% of patients on PSMA-PET/CT missed by CT and BS [75].

## Long-term follow-up and progression monitoring

### PSMA PET/CT

PSMA-PET/CT can act as a prognostic tool for long-term response assessment—Grubmüller et al found TTV measured using modified PERCIST to correlate with PSA response and OS, whereas conventional RECIST1.1 criteria based on anatomical tumour size did not reliably predict outcomes. This highlights the role of volumetric tumour burden as a more sensitive measure of survival compared to morphology-based criteria [76]. This was further supported by the LuPIN trial, with increases in TTV predicting early progression and OS [77]. However, neuroendocrine differentiation is a known cause of false-negative PSMA-PET/CT findings due to loss of PSMA expression. In these cases, additional imaging with [¹⁸F]FDG-PET/CT may be helpful, although the optimal timing for this has not been defined [58].

Beyond prognostication, PSMA-PET/CT is the primary tool for detecting disease recurrence due to its high sensitivity and specificity. There is currently no standardised protocol for the frequency of repeat imaging, but scans may be performed either at regular intervals or when biochemical or clinical suspicion of progression arises.

### ctDNA dynamics

ctDNA provides a non-invasive means of assessing tumour burden in real time. Analysis from PSMAfore found that patients with 8q amplifications, AR amplifications, and TP53 deleterious alterations were associated with shorter rPFS and decreased tumour response in the [^177^Lu]Lu-PSMA-617 arm [78]. Monitoring early ctDNA changes could act as an early signal of response prior to imaging results. Methylated-ctDNA, such as KLF8, AKR1B1 and LDAH, have been shown to follow response dynamics, with SHOX2 and SEPT9 correlating with imaging changes during therapy [79, 80].

However, ctDNA sensitivity is dependent on tumour burden and shedding rates; therefore, patients with low-volume disease may release insufficient ctDNA for reliable detection. Additionally, there is no clear standard for defining progression or for sample collection, which limits clinical interpretation. The limited availability of validated assays and infrastructure also restricts the integration of ctDNA monitoring into clinical practice. Nevertheless, ctDNA is a promising complementary tool for early detection of treatment failure and refined response assessment.

## Other emerging techniques

### Imaging

#### WB-MRI

Whole-body MRI is a radiation-free imaging alternative, offering both morphological and functional tumour assessments. Additionally, sequences such as T1/T2 and relative Fat Fraction (rFF%) enable accurate tumour sizing to support RECIST1.1 reporting [81].

Whole-body diffusion-weighted MRI (WB-DWMRI) is a non-irradiating alternative, offering high soft-tissue contrast and spatial resolution, well-suited for detecting ‘all-organ’ PCa lesions. WB-DWMRI outperforms bone scintigraphy in detecting bone metastases, and matches CT in identifying enlarged lymph nodes, suggesting its potential to replace conventional multimodal staging approaches [17].

Quantitative rFF% maps from T1-gradient echo Dixon images and apparent diffusion coefficient (ADC) metrics can characterise lesions and assess response [82]. Inter-reader agreement is strong for most regions (κ = 0.61–1.0), except pelvic nodes (κ = 0.56), with excellent repeatability for global ADC metrics, e.g., ADC_mean_, ADC_median_ and tumour diffusion volume (ICC = 0.97–0.99), essential for consistent response evaluation [83, 84].

#### Patient selection

As up to 10% of PCa lesions do not overexpress PSMA, WB-DWMRI offers an advantage in PSMA-negative disease. It allows whole-body tumour burden assessment and tailoring of salvage treatments in biochemical recurrence, characterises lesion activity by signal intensity, and detects liver metastases (prognostically worse than nodal-only disease) [82, 85]. Nieuwenhove et al achieved an acquisition time of 29 min, and found WB-MRI to detect primary lesions in 5 additional patients vs. [^68^Ga]Ga-PSMA-11 PET/CT, 3 of whom had previous TURP–potentially from lack of masking effects from radiotracer accumulation. WB-MRI also outperformed PET/CT in detecting local extension in 64% vs. 54% of patients [86].

Bone marrow imaging is a key strength of WB-DWMRI: active bone metastases show high signal on high b-value DWI, low-intermediate ADC, and low signal on rFF% and T1-weighted sequences [82]. Lower rFF% correlates with malignancy and poorer prognosis, and ADC volume correlates with cancer-specific response [85]. Donners et al demonstrated that combining DWI signal, ADC < 1100 μm²/s, and rFF% < 20% yielded 80% sensitivity and 93% PPV for identifying biopsy-positive lesions [87].

Affected nodes are identified by size criteria (short axis > 10 mm/> 8 mm for pelvic nodes), or abnormal morphology (irregular contours, loss of fatty hilum, deviation from normal “kidney-shape”) [82]. However, smaller nodes may be missed: sub-8mm nodes account for 74% of malignant nodes. In primary PCa, ^18^F-PSMA-PET/CT detected 83% of malignant nodes, compared to 58% with WB-DWMRI [88]. ADC values between benign and pelvic nodes differ significantly, aiding in differentiation [87].

Although not yet evaluated directly, WB-DWMRI may predict responses to [^177^Lu]Lu-PSMA-617 therapy: ADC values inversely correlate with Gleason score, akin to FDG-PET, which has shown promise in patient selection from trials such as TheraP. WB-DWMRI offers greater anatomical clarity than FDG-PET/CT, and combining ADC with PSMA-PET SUV could enable comprehensive tumour characterisation and aggressiveness.

#### Response assessment

The METastasis Reporting and Data System for Prostate Cancer (MET-RADS-P) standardises lesion response assessment through MRI signal intensity, whilst accounting for discordant responses at the regional level [89]. WB-DWMRI can detect response quantitatively (reduced lesion size, decreased signal intensity on high b-value DWMRI, and increased ADC), as well as qualitatively (diffuse disease returning to fatty marrow) [90]. A ‘fatty halo’ on T1-weighted MRI indicates response, whilst a ‘cellular halo’ suggests progression [82]. MRI also detects skeletal-related events (e.g., fractures, cord compression), relevant for progression assessment [82]. However, post-therapy changes and PSMA flares complicate interpretation.

In a cohort of 53 patients assessing PCa recurrence after local treatment, WB-MRI achieved 98–100% sensitivity and specificity for bone metastases, 77–82% sensitivity and 96–98% specificity for lymph nodes, and 60% sensitivity and 100% specificity for visceral lesions [17]. Decreased volume of bone metastasis was associated with higher FF, and responding bones had variable ADC due to increased fat infiltration [91]. Blackledge et al investigated semi-automated segmentation techniques to distinguish between responders (increased total diffusion volume) from non-responders (larger increase in global ADC) post-chemotherapy. A similar approach may be applicable for ^177^Lu-PSMA-Therapy [92].

Despite its strengths, WB-DWMRI has limited sensitivity for sub-8mm nodes, and EAU guidelines recommend PSMA-PET/CT to detect biochemical recurrence [82]. Sawicki et al found WB-DWMRI missed PSMA-avid nodal metastases (but considered radiologically benign) compared to PET/CT in patients with documented biochemical recurrence following radical prostatectomy [93]. Hybrid PSMA-PET/MRI has outperformed PSMA-PET/CT in local recurrence detection at lower PSA levels, particularly in detecting local recurrences in the prostate bed due to the MRI component [94].

### SSTR PET/CT

One recognised mechanism of treatment resistance is through loss of PSMA expression and acquisition of neuroendocrine characteristics, including upregulation of somatostatin receptors, chromogranin, and synaptophysin. This can be detected with SSTR-PET, in combination with ^18^F-FDG and PSMA-PET/CT, for a more comprehensive evaluation of tumour heterogeneity and underlying biology, which may not be apparent through PSA levels or PSMA-imaging alone [95].

This multimodal approach was exemplified in the prospective Triple-Tracer strategy against Metastatic PrOstate cancer (3TMPO) study, which utilised all three tracers to assess intermetastatic intrapatient heterogeneity (IIH) in mCRPC: IIH was detected in 82.7% of patients, with 45.9% demonstrating at least one discordant lesion that was FDG+ve/PSMA-ve. Notably, 6% of patients had entirely FDG+ve/PSMA-ve disease, consistent with the 10% reported in the TheraP trial. This subgroup likely represents a distinct and aggressive tumour biology that is unsuitable for PSMA-RLT. Moreover, SSTR-PET findings carried independent prognostic significance, as those with at least one [^68^Ga]Ga-DOTATATE-positive lesion had reduced OS. Iravani et al reported that none of the patients with CRPC NED were eligible for SSTR-directed therapy, reinforcing the prognostic rather than therapeutic value of SSTR-PET [96]. The tumour sink effect was also demonstrated in this study, with patients with a large tumour burden (widespread/intensely avid metastases) receiving lower healthy tissue irradiation. A more personalised approach, incorporating tumour burden, body habitus, and renal function, could allow for higher administered activities in heavily burdened patients, potentially improving outcomes [97].

### Dosimetry

Dosimetry has a role in the quantitative assessment of radiation delivery to metastatic sites and normal tissues. Violet et al found that patients absorbed doses < 10 Gy to tumour sites at 12 weeks were less likely to achieve PSA50, and a potential biomarker to guide treatment intensity [98].

Extrapolation of cumulative absorbed doses has found cycle 1 dosimetry data to acceptably estimate cumulative doses over multiple cycles [99]. Whilst single-time-point absorbed dose estimates offer reduced imaging times, not all studies have found a correlation between absorbed doses and response [67, 100]. Utilising multi-time-point serial SPECT/CTs captures the temporal kinetics and improves the accuracy of dose estimates. Kinetic analyses have demonstrated that responders typically exhibit stable or slowly decreasing tracer activity within metastases, whereas non-responders show activity reduction within the metastases. These patterns may reflect differences in intratumoral residence time and could serve as early prognostic indicators for both PFS and OS [101].

Monte Carlo simulations are the gold standard for dosimetry, as they account for patient-specific anatomy and tissue composition derived from CT imaging [100]. Other alternatives include voxel-based dosimetry, which can achieve dose estimates within 6% of Monte Carlo simulations. In contrast, traditional organ-based methods such as OLINDA/EXM had dose errors of 123%, especially in bone marrow or bone metastases, where heterogeneity is pronounced [102].

Dosimetry can facilitate personalised activity planning, ensuring delivery of maximal tumouricidal radiation while sparing organs at risk such as kidneys (due to PSMA expression in proximal tubular cells and urinary excretion). Similarly, the salivary and lacrimal glands are prone to radiotoxicity, which may lead to xerostomia and dry eyes [99]. In such settings, post-therapy dosimetry can guide dose adjustments or supportive care strategies. Beyond acute toxicity, long-term monitoring of cumulative organ doses is essential to detect delayed adverse effects. While the risk of therapy-related myeloid neoplasms appears low (1.3% incidence), it remains a concern as RLT is adopted earlier [103]. By combining kinetic modelling and patient-specific anatomical data, it is increasingly possible to tailor RLT to individual tumour biology and organ tolerance.

### Radiomics and AI

The number of PSMA-positive lesions on ^68^Ga-PSMA-PET/MRI correlates with higher PSA levels and shorter PSA doubling time [104]. Roll et al extracted radiomic features from PET-positive tumour volumes on WB-MRI and developed a logistic regression model using T2-weighted MRI, achieving 0.83 area under the curve for predicting biochemical response. Ten radiomic features differentiated responders from non-responders [105].

AI application in PSMA-PET/CT includes prognostication through the detection of primary tumours, tumour quantification, and radiomic feature extraction. Semi-automated software segments primary and metastatic lesions to derive metrics such as TL-PSMA, PSMA-TV and SUV [106]. Fully automated systems can detect lesions with 87–94% accuracy and 88–95% sensitivity, extract biomarkers such as TL-PSMA and PSMA-TV with > 90% accuracy and sensitivity, and detect disease recurrence [107]. These approaches can predict PSA-PFS and OS [108]. AI-based lesion tracking enables comprehensive assessment of numerous metastatic sites, capturing intermetastatic heterogeneity and treatment response. This is important in advanced PCa, where multifocal and heterogeneous disease can markedly influence outcomes [109].

ML can be applied to risk stratification and individualised dosimetry: Yazdani et al combined pre-therapy [^68^Ga]Ga-PSMA PET/CT radiomics with clinical biomarkers to predict absorbed doses in kidneys and tumours [110]. Complementary deep learning frameworks have also generated post-treatment PSMA-PET images from baseline scans, accurately reproducing radiotracer uptake patterns and predicting lesion response [111].

AI also supports the evaluation of bone involvement, with tools such as EBONI quantifying metastatic bone burden on PSMA-PET/CT in under 3 min [112]. ML-based CT texture analysis distinguishes metastatic from fully responded sclerotic lesions on post-therapy [^68^Ga]Ga-PSMA-11 PET/CT [113].

Generative AI approaches have the potential to simulate high-quality PET images from low-dose scans, synthetically generate labelled images to train AI models (minimising false outcomes), and perform cross-tracer adaptation (e.g., ⁶⁸Ga→¹⁸F). These tools can also capture intra-tumoural and interorgan heterogeneity of PSMA uptake, further supporting individualised treatment decisions [114]. Beyond quantification, ML models using pre-therapy PET/CT imaging and lab data can predict organ-specific radiation doses more accurately than population-based methods, potentially reducing haematologic toxicity by tailoring treatment plans [115, 116]. Furthermore, advances such as total-body PET/CT or long axial-field-of-view PET/CTs enhance lesion detection through greater anatomical coverage, increased sensitivity, and increased target lesion signal-to-noise ratio compared to PSMA-PET/CT [117]. Machine learning has enabled ultra-low-dose CT reconstruction and ultra-fast PET acquisition, maintaining diagnostic quality while reducing scan time by up to 40-fold, but remains limited in diagnostic sensitivity compared to standard PSMA-PET/CT [118].

### Novel biomarkers

Amplifications in FGFR1 and CCNE1 occur more frequently in non-responders and likely reflect underlying genomic instability [119]. These are accompanied by increased copy number variation burden, linked to shorter PFS and OS [120]. Tumour suppressor gene alterations in TP53, RB1, and PTEN are independently associated with poor outcomes following PSMA-targeted radioligand therapy [121, 122]. Defects in homologous recombination repair genes and androgen receptor alterations correlate with higher mean PSA and shorter duration of response [123]. Cyclin-dependent kinase 12 (CDK12) mutations predict poor outcomes and raised non-responders [119]. At the pathway level, dysregulation of the PI3K/AKT/mTOR axis through PTEN loss is an adverse prognostic marker [121, 122].

## Prognostic/predictive integration

### Composite models

Several prognostic models have integrated clinical and imaging biomarkers to guide selection for ^177^Lu-PSMA-Therapy. Gafita et al proposed nomograms predicting rPFS and OS combining variables (such as time from initial diagnosis, baseline haemoglobin level) with PSMA-PET/CT derived parameters [124]. These models were validated in a randomised setting and showed reduced accuracy in the validation cohort relative to the original. Among these, the PSA50 model demonstrated predictive utility: decision curve analysis identified clinical benefit when applied to patients with a ≥ 30% model-predicted probability of PSA response, supporting its role in guiding the choice between [¹⁷⁷Lu]Lu-PSMA-617 and cabazitaxel. These findings establish the PSA50 model as both a prognostic and predictive biomarker, whereas OS and PSA-PFS models remain prognostic only [125]. A VISION post-hoc analysis utilised SUV_max_ and PSMA-positive nodes from [^68^Ga]Ga-PSMA-11 PET/CT, and blood parameters to predict OS and rPFS [126]. These models have potential utility as clinical decision support tools, helping integrate imaging and clinical data to personalise selection for ^177^Lu-PSMA-Therapy, and sparing non-responders unnecessary toxicity.

### Machine learning

Moazemi et al developed a deep learning model to predict responders vs. non-responders to [^177^Lu]Lu-PSMA-617 therapy using clinical information and baseline imaging. Baseline [^68^Ga]Ga-PSMA PET/CT radiomics were combined with serum alkaline phosphatase (ALP) at first PSMA-PET/CT, diagnosis-to-scan interval, and Gleason score. This achieved 0.8AUC (75% sensitivity and specificity), demonstrating potential as a decision support tool [127]. A later model used recursive feature elimination to identify 14 features and generated a fully automated framework for response prediction [128]. However, using radiomic features (sensitive to acquisition and reconstruction variability) may introduce spurious patterns and increase the risk of type-I errors.

Gong et al integrated pre-therapy imaging with serum biomarkers (such as ALP, neutrophil, and leucocyte counts) to distinguish patients likely to achieve full therapeutic benefit from those with suboptimal outcomes, achieving a predictive accuracy of 0.92 and sensitivity of 0.96. High renal choline uptake, abnormal blood parameters, and PSMA-FDG mismatch were associated with poor treatment response, highlighting their utility in patient selection and therapeutic optimisation [129].

## Challenges

Implementing WB-MRI protocols across diverse MRI hardware/software platforms highlights the need for standardisation, essential for quantitative analysis. False positives can arise from fat-water swap artefacts on Dixon images or from marrow reconversion mimicking metastases, and incidental findings are common, requiring further clinical investigations, potentially overwhelming already stretched services [17]. Acquisition time (typically 40–60 min) limits widespread use. Emerging deep learning-based denoising filters may reduce this to 5 min by improving image quality from sub-sampled data [130]. Similarly, variations in PET and SPECT acquisition and reconstruction protocols can lead to inconsistencies in quantitative parameters and dosimetry estimates. Without standardised quantitative metrics, cross-trial comparisons and radiomic feature reproducibility are limited. Establishing guidelines for PET/SPECT quantification and radiomic feature extraction will be essential for personalised RLT.

## Future directions

Radiomics and AI models show promise for predicting treatment response, personalising dosimetry, and refining patient selection. However, most models are retrospective, single-centre, and based on relatively small datasets, raising concerns about overfitting and limited generalisability. Prospective, multicentre trials are needed to confirm predictive performance and to establish reproducibility across different protocols and patient populations.

PCWG4 is an emerging standard, integrating serial PSMA-PET/CT instead of BS into response and progression assessment as a more sensitive tool for monitoring disease. Preliminary criteria were tested using data from the PRINCE trial, showing substantial inter-reader agreement for response levels and progression detection (κ = 0.90). Importantly, PCWG4 criteria identified progression earlier than PCWG3 (median rPFS 9.4 vs. 19.9 months) and correlated strongly with OS, suggesting that PSMA-PET/CT may offer a more timely evaluation of treatment efficacy [131].

In parallel, the Standardised PSMA Assessment and Reporting Consensus (SPARC) initiative is working to standardise PSMA-PET interpretation and reporting. Key updates include consolidation of prior reporting systems (PRIMARY score, PROMISE/miTNM, PSMA-RADS, PSMA-VOL), reporting tumour metrics for PSMA expression, tumour volume, SUV_mean_/region-specific SUV_max_, and lesion count, and a 5-point Likert scale for diagnostic certainty. Both PCWG4 and SPARC are looking to provide a standardised framework for supporting clinical decision making [132].

## Conclusion

PSMA-PET/CT is the established standard for selecting patients for ¹⁷⁷Lu-PSMA-therapy, whilst PSA monitoring, CT, and BS under PCWG3 remain standard for assessing response and progression. Post-therapy SPECT/CT is used for dosimetry and early prognostic assessment, but its use is not yet standardised across centres. Quantitative PSMA-PET metrics, ctDNA analysis, WB-DWMRI, and personalised dosimetry are emerging tools with potential to improve response evaluation and therapy planning. Additional approaches, including SSTR-PET/CT, radiomics, AI/ML for lesion quantification and adaptive dosimetry, and novel genomic biomarkers, may further personalise treatment. New frameworks such as PCWG4 and SPARC aim to standardise PSMA-PET reporting and response assessment, reflecting a shift toward multimodal and individualised strategies for selecting and monitoring ¹⁷⁷Lu-PSMA-therapy.

## Data Availability

Please see references.

## Abbreviations

ALP: Alkaline phosphatase
ARPI: Androgen receptor pathway inhibitors
IIH: Intermetastatic intrapatient heterogeneity
mCRPC: Metastatic castrate resistant prostate cancer
OS: Overall survival
PCa: Prostate cancer
PCWG3/PCWG4: Prostate Cancer Working Group 3/4
PERCIST: PET response criteria in solid tumours
PFS: Progression-free survival
PI3K/AKT/mTOR: Phosphoinositide 3-kinase/protein kinase B/mammalian target of rapamycin
PPP: PET progression criteria in prostate cancer
PROMISE V2: Prostate Cancer Molecular Imaging Standardised Evaluation version 2
PSMA-TV: PSMA-avid tumour volume
RECIP 1.0: Response Evaluation Criteria in PSMA Imaging Prostate Cancer Working Group 1.0
RLT: Radioligand therapy
SOC: Standard of care
SPARC: Standardised PSMA assessment and reporting consensus
SUV: Standard uptake volume
TL-PSMA: Total lesion PSMA
TTV: Total tumour volume
WB-DWMRI: Whole-body diffusion-weighted MRI
